# Ultraviolet Radiation, Aging and the Skin: Prevention of Damage by Topical cAMP Manipulation

**DOI:** 10.3390/molecules19056202

**Published:** 2014-05-15

**Authors:** Alexandra Amaro-Ortiz, Betty Yan, John A. D’Orazio

**Affiliations:** 1 The Graduate Center for Toxicology, the Markey Cancer Center and the Department of Pediatrics, University of Kentucky College of Medicine, 800 Rose Street, Lexington, KY 40536, USA; 2 Markey Cancer Center, University of Kentucky College of Medicine, Combs Research Building 204, 800 Rose Street, Lexington, KY 40536-0096, USA

**Keywords:** forskolin, aging, UV radiation, skin, oxidative stress

## Abstract

Being the largest and most visible organ of the body and heavily influenced by environmental factors, skin is ideal to study the long-term effects of aging. Throughout our lifetime, we accumulate damage generated by UV radiation. UV causes inflammation, immune changes, physical changes, impaired wound healing and DNA damage that promotes cellular senescence and carcinogenesis. Melanoma is the deadliest form of skin cancer and among the malignancies of highest increasing incidence over the last several decades. Melanoma incidence is directly related to age, with highest rates in individuals over the age of 55 years, making it a clear age-related disease. In this review, we will focus on UV-induced carcinogenesis and photo aging along with natural protective mechanisms that reduce amount of “realized” solar radiation dose and UV-induced injury. We will focus on the theoretical use of forskolin, a plant-derived pharmacologically active compound to protect the skin against UV injury and prevent aging symptoms by up-regulating melanin production. We will discuss its use as a topically-applied root-derived formulation of the *Plectranthus barbatus* (*Coleus forskolii*) plant that grows naturally in Asia and that has long been used in various Aryuvedic teas and therapeutic preparations.

## 1. Introduction

**Figure 1:**
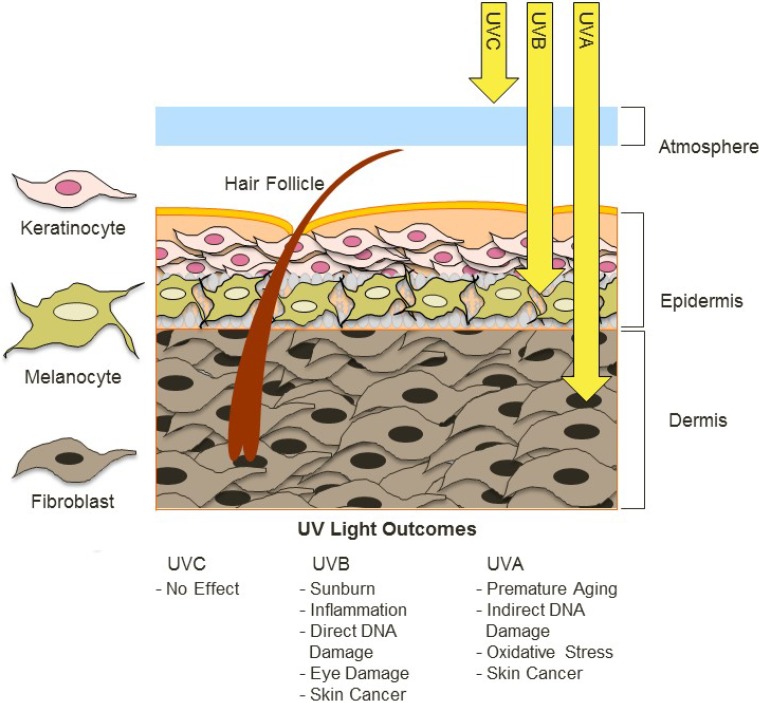
UV radiation in ambient sunlight is composed primarily of UVA and UVB energy. Most UVC is absorbed by the ozone, therefore although it is highly bioactive, terrestrial organisms are not exposed to significant levels of UVC. UVB can cause direct damage to DNA and reach the epidermis. UVA can penetrate the dermis and increases levels of ROS that indirectly induce DNA mutagenesis.

Due to its anatomic location at the external boundary of the body, skin is exposed to a variety of environmental factors such as UV radiation that derives naturally from the sun. Solar UV exposure is a major causative factor for age-related changes such as skin cancer development. UV radiation is composed of UVA, UVB and UVC components based on photon wavelength with UVA having the longest wavelengths (315–400 nm), UVB being mid-range (290–320 nm) and UVC being the shortest wavelengths (100–280 nm). Ambient sunlight is composed primarily of UVA (90%–95%) and UVB (5%–10%) energy, with most solar UVC absorbed by the ozone layer (Figure 1).

Naturally occurring UV radiation is the environmental mutagen responsible for the largest percentage of environmentally induced skin pathologies, including erythema and inflammation, degenerative aging changes, and cancer [1]. Humans are exposed to UV radiation primarily as a consequence of unprotected exposure to sunlight [2]. UV radiation has many deleterious effects on cells [3,4,5]. UV radiation produces both direct and indirect DNA damage, and each can result in mutagenesis in skin cells. The DNA double helix can absorb energy from shorter-wave UV photons and undergo covalent modification. Neighboring pyrimidines are particularly vulnerable to direct UV damage at the 5–6 double bond position. When UV causes this bond to break, abnormal covalent interactions between adjacent thymines and/or cytosines can result. There are two main DNA lesions that result from UV-induced damage to the 5–6 double bond: (1) cyclobutane dimers formed from two covalent bonds between adjacent pyrimidines to form a ring structure; and (2) pyrimidine 6–4 pyrimidone (6,4)- photoproducts that form upon reaction of the open 5–6 double bond with the exocyclic moiety of an adjacent 3' pyrimidine [6]. Both of these lesions distort the double helix and can lead to mutation, and an individual skin cell may accumulate up to 100,000 such lesions from one day’s worth of sun exposure [7]. UV radiation also damages cellular macromolecules indirectly, through production of oxidative free radicals [8]. Several DNA modifications can result from oxidative injury, including 7,8-dihydro-8-oxoguanine (8-oxoguanine; 8-OH-dG), which promotes mutagenesis (specifically GC-TA transversion mutations [9]. Both direct and indirect DNA changes interfere with transcription and replication, and render skin cells susceptible to mutagenesis. Much of solar UV energy is absorbed by stratospheric ozone, and the gradual depletion of stratospheric ozone over the last several decades has resulted in higher levels of solar UV radiation that strikes the surface of the Earth [10]. Increased ambient UV radiation from global climate change may be an important factor to explain the burgeoning prevalence of melanoma and skin cancer over the last several decades [11,12,13,14].

UVB is a well-characterized mutagen and inducer of skin cancers [15], but recent studies have implicated an increasing role of UVA as a carcinogen [16,17,18] likely through its pro-oxidative effects and possibly through other mechanisms such as telomere shortening [19]. In addition, UVA is less able to induce melanin production compared to UVB, leaving the skin less able to protect itself against further UV insult [15,19,20,21,22]. Increasing attention is being paid to the potential impact of UVA radiation to the body focusing on differential cellular repair and apoptosis depending on anatomic site [23]. The role of UVA in melanoma formation is also suggested by the observation of rising melanoma incidence over the last several decades and sunscreen use in the 1980s when only UVB-blocking sunscreens were used.

## 2. Factors Contributing to UV Exposure

Geographical variations such as altitude, latitude and urbanization all determine ambient UV strength. Because atmospheric particles such as dust or water droplets can scatter, reflect or otherwise interfere with UV photons, the more atmosphere sunlight has to traverse, the weaker its energy will be at the surface of the Earth. At higher altitudes, with less atmosphere for sunlight to traverse before hitting land, there is higher exposure to UV and a higher risk for melanoma. There is a 2% increase in risk with every 10 m rise in altitude [24] and in addition those living at altitudes over 1,400 m above sea level are at most risk for developing melanoma [25]. In addition to living in high altitudes, occupations routinely operating at high altitudes such as airplane pilots and mountain guides have a higher incidence of melanoma and precancerous lesions [26,27].

UV strength is strongest at the equator because sunlight hits the Earth most directly at the equator. Toward the poles, sunlight hits the earth obliquely and must pass through more atmosphere. Not surprisingly, there is a higher incidence of melanoma in locales closest to the equator, most especially among Caucasians [28] who are most UV-sensitive because of lower cutaneous melanin pigments, but also among lower-risk populations of darker skin tone [29]. In a study of the Norwegian Cancer Registry, decreased latitude by 10° was associated with a 2–2.5 increased risk of melanoma [30]. Another study of 5,700 melanoma cases worldwide found a 1.5-fold increased risk when living at latitudes closer than 20° from the equator [31]. Importantly, though latitude risk for melanoma has been historically strong, recent studies suggest decreased correlation [32], or even an opposite trend. A 2012 study of Northern Europeans, for example, showed an increase in melanoma incidence with increase in latitude beyond 50° north of the equator [33], perhaps due to the dramatic rise in artificial indoor tanning.

Urban-versus-rural lifestyle also seems to be important, with as much as a 50% increased melanoma risk in urban regions [34]. Urbanization may affect cancer risk by bringing together many independent risk factors such as occupational chemical exposure, social pressures regarding skin appearance, easy access to indoor tanning, and higher socio-economic levels lending to increased use of indoor tanning and holiday travel [35]. The increase in urbanization worldwide and the increase in these activities may help explain the rise in melanoma rates for northern Caucasian populations that would otherwise not be exposed to natural risk factors such as latitude [24].

## 3. Age

UV exposure may account for up to 80% of visible signs of aging in the skin including dry appearance, scalping, wrinkling [15] and impaired pigmentation, and photoaging correlates with cancer risk. A 2012 study of Central Europeans, for example, showed those with early signs of wrinkling on the neck were over four times more susceptible to melanoma than the general population. Freckling on the back also showed over three times the risk [21]. Cutaneous photoaging and melanoma risk both correlate with age and UV exposure. The average age of melanoma diagnosis is about 55 and incidence varies worldwide from five to over 60 cases per 100,000 people per year [12]. Although melanoma is a malignancy mostly diagnosed in the fifth and sixth decade of life, one fifth of cases occur in young adults [36,37]. It is important to note, however, that the UV exposure and accumulation of DNA damage that underlie melanoma formation begin with sun exposure early in youth, which is why sun protection in the pediatric years is so important. There is a significant correlation of melanoma risk with excessive sun exposure before adolescence, perhaps contributed to by structural anatomical differences between the skin of children and adults making it easier for UV to penetrate [38]. Childhood UV exposure also increases the risk of young adult melanoma (melanoma under the age of 30) by over three times, showing how exposure can accelerate the process of carcinogenesis [39]. Furthermore, a new study published in 2014 of over three million people in Sweden showed that accumulation of UV damage begins as early as in the neonate, with melanoma incidence increased in those born in the spring and summer *versus* those born in the fall or winter [40]. Indeed, some estimates indicate that up to 80% of lifetime UV exposure occurs before the age of 20 because of the outdoor recreational habits of children.

This risk for melanoma among the middle-aged population has risen in the past few decades. An epidemiologic study in Minnesota found an incidence of 60 cases per 100,000 in 2009 compared to just eight per 100,000 in 1970; that is a 24-fold increase in risk for this population. Another unfortunate finding is the steady increase in occurrence in young adults, particularly for young women in the United States (US). Whereas young American women aged 15–39 had a melanoma incidence rate of 6 out of 100,000 cases in 1973, their rate more than doubled to 14 out of 100,000 cases per year in 2006 [41]. Because of ongoing recreational UV trends such as increased use of artificial tanning sources, melanoma rates are expected to continue to rise [37], making this disease an increasing public health threat.

## 4. Artificial UV and Tanning Beds

Indoor tanning use has dramatically risen in the last thirty years and is predicted to continue rising largely because of societal and commercial incentives for a tanned appearance viewed by many as appealing. In 2013, over 40% of adolescents aged 15 to 18 had tried indoor tanning with about 18% using indoor tanning routinely [42]. A recent large-scale systematic review, published in 2014, reported that over 50% of college-aged students tried indoor tanning, with over 40% using it in the past year. In a 2014 study of college-aged women from 18 to 25 years of age, 25% of current users could be classified as tanning-dependent [43]. Similarly, prevalence among American adults may be as high as 35% [42,44]. Interestingly, tanning behavior has increasingly been compared to classic “substance use” disorders, with some classifying frequent indoor UV patronage as a true addiction [45,46]. Tanning-addictive behaviors have been associated especially with young age, other high-risk behaviors and psychiatric disorders [45,46]. Indoor tanning involves exposure to high doses of UV with the intent to trigger skin pigmentary responses. Tanning beds emit varying blends of UVA and UVB energy, and their use is clearly linked with photoaging, keratinocyte malignancies and melanoma. Generally, most basic tanning beds emit a blend of UVB and UVA radiation with more advanced beds emitting mostly UVA radiation to emulate natural UV radiation. However, with UVA now firmly implicated in melanoma carcinogenesis, such beds may be no safer than those with higher UVB output. Alarmingly, despite strong epidemiologic data correlating younger age of tanning bed use with skin cancers, tanning bed use among minors is poorly regulated and its use increasing. In 2013, the National Conference of State Legislatures of USA approved new laws limiting or banning the use of tanning beds by adolescents, however actual laws that govern indoor tanning among minors vary by state with most states not prohibiting use among minors. Use of tanning salons before the age of 35 years is associated with a 75% increased lifetime melanoma risk [47], therefore the increasing use of tanning beds may be an important factor to explain the increasing incidence of melanoma in recent decades.

## 5. Sunburns and Melanoma

Overexposure to UV is a key factor in development of skin cancers, and melanoma incidence correlates particularly with intermittent intense UV exposures that cause sunburn. More than five sunburns in a lifetime doubles risk for melanoma and there is increased risk for melanoma as a young adult if there are increased sunburns in childhood [31,39,48]. The melanoma-sunburn link may reflect the contribution of inflammatory mediators to carcinogenesis or perhaps a particular threshold above which the dose of UV must exceed in order to transform melanocytes. Nonetheless, intense blistering sunburns seem to play a role in many cases of melanoma. Unfortunately, over half of all adults in the US suffered from sunburn in 2013 and prevalence of sunburn in the US population today is over 50% in all adults and over 65% in fair-skinned young adults under the age of 30. Furthermore, prevalence of sunburn has not declined despite the variety of lotions, sprays and clothing advertised and available for sun protection [49]. Risk of sunburn is complex and influenced by a variety of factors ranging from geography, cloud cover, climate, societal norms relating to amount of clothing worn, *etc.* Not surprisingly, incidence of sunburns in children (and indoor tanning use among adolescents) correlates with parental attitudes regarding sun protection. For this reason, educational campaigns targeted at parents’ ideas about UV safety might be particularly useful for prevention and protection against UV-induced skin pathologies [50].

## 6. The Melanocortin 1 Receptor (MC1R) and the Tanning Response

**Figure 2:**
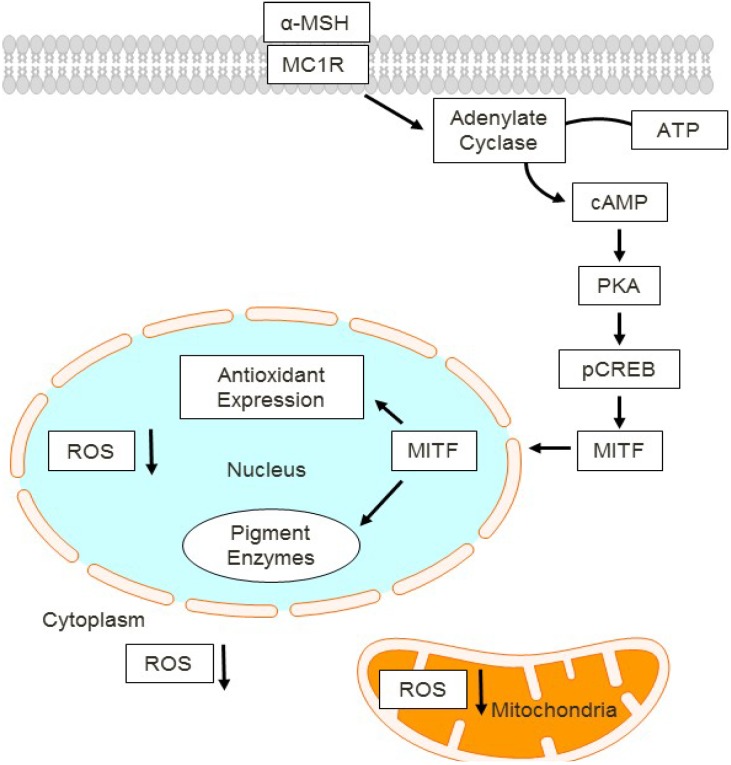
MC1R signaling cascade in melanocytes. Activated by its agonist alpha-MSH, MC1R promotes cAMP second messenger generation which induces melanocyte differentiation and survival pathways involving PKA, CREB and Mitf. In this way, cAMP induces both melanin production and antioxidants that reduce cellular ROS. cAMP, cyclic adenosine monophosphate. PKA, protein kinase A. pCREB, phosphorylated cAMP response binding element. ROS, reactive oxygen species. MITF, microphthalmia (Mitf) transcription factor.

One important aspect of the solar radiation in human’s skin is the adaptive tanning response. After UV exposure, cellular damage response activation induces melanin production by melanocytes and proliferation and melanin deposition in keratinocytes, all of which result in enhanced pigmentation of the skin. This important physiologic pathway is a natural UV-protective response to protect the skin against further UV insult after an initial UV exposure. The ability of the skin to tan depends on the function and activity of the cutaneous melanocortin 1 receptor (MC1R) signaling pathway [51,52,53,54,55] (Figure 2).

The MC1R is a G_s_-couple protein located in the extracellular membranes of epidermal melanocytes. When bound by agonistic ligands, most notably α-melanocyte stimulating hormone (α-MSH) [51], the MC1R initiates a cascade of UV-protective events mediated by activation of adenylyl cyclase and generation of the second messenger cAMP. MC1R activates adenylate cyclase that converts ATP to cAMP which activates protein kinase A (PKA). PKA phosphorylates the cAMP-responsive binding element (CREB) and induces activation of the microphthalmia (MITF) transcription factor. MITF is a myc-like master transcription factor that, in melanocytes, drives expression of tyrosinase and other pigment biosynthetic enzymes. In this way, epidermal melanocytes produce melanin pigment that gets deposited in the epidermis to physically interfere with penetration of UV photons, thereby protecting skin cells from the damaging effects of sunlight [56]. Importantly, MC1R signaling also influences the ability of melanocytes to recover from UV-induced DNA damage [57,58,59,60,61]. Overall, there is much evidence placing MC1R as a “master regulator” of melanocyte UV physiologic responses.

## 7. Pigmentation Phenotype Depends on MC1R Signaling

Loss-of-function polymorphisms of MC1R lead to a fair-skinned, sun-sensitive, and cancer-prone phenotype [62,63,64]. The major MC1R polymorphisms among human populations are the so-called “red hair colored” (RHC) genotypes that yield a characteristic UV-sensitive and melanoma-prone phenotype, namely propensity for sun burning rather than tanning, fair skin complexion, freckling and red/blonde hair. The RHC phenotype include R151C, R160W and D294H MC1R genotypes [65,66,67]. In these cases, there is blunted cAMP production in melanocytes, and the skin produces less of the highly UV-protective dark brown/black pigment species known as eumelanin. Instead, there is production of a red/blonde pigment known as pheomelanin that is much less effective at blocking incoming UV energy and may even potentiate UV-induced oxidative injury. Without effective cAMP induction in melanocytes, as is the case in MC1R-defective individuals, the skin cannot accumulate significant amounts of eumelanin and therefore will be prone to UV damage and carcinogenesis.

## 8. Forskolin Rescues cAMP Deficient Signaling

Forskolin is a naturally derived diterpenoid extracted from the roots of the *Plectranthus barbatus* (*Coleus forskolii*) plant that grows naturally in Asia and that has long been used in various Aryuvedic teas and therapeutic preparations. Forskolin, which is a skin-permeable compound, directly activates adenylate cyclase to induce production of cAMP. Our laboratory was among the first to show that topical application of forskolin promoted UV-independent production of eumelanin in an MC1R-defective fair-skinned animal model [53], resulting in robust UV protection by interfering with epidermal penetration of UV photons [68]. Pharmacologic stimulation of cAMP using forskolin may protect the skin in ways other than through melanin induction. For example, cAMP provided enhancement of keratinocyte migration to promote wound healing [69] and it also decreased blister formation [70]. De Vries and co-workers proposed using a topical cAMP approach to regulate beta-adrenergic response in psoriasis patients [71]. Interestingly, cAMP stimulation has also been studied as an activator of hair follicle activity and has been considered as a therapy for age-related hair loss [72,73]. We and others have been interested in the UV-protective consequences of topical cAMP induction to promote melanin protection from UV-mediated DNA damage [68] and to enhance levels and/or activity of key DNA repair and antioxidant enzymes [74]. Forskolin and other cAMP-promoting agents may also protect the skin against UVB-induced apoptosis [75] and by promoting epidermal thickening which also aids in resisting UV damage [76]. In particular, Scott *et al.* reported that cAMP-mediated accumulation of basal and epidermal keratinocytes resulted in a melanin-independent mechanism of blocking UVA and UVB penetration into the skin [76]. Others reported that forskolin protected against generation of oxidative stress by decreasing levels of nitric oxide [77] and enhancing stimulation of the cytoplasmic antioxidant enzyme copper/zinc superoxide dismutase (Cu/ZnSOD) [78]. Taken together, studies suggest that pharmacologic induction of cAMP in the skin may represent a potential UV-protective strategy for MC1R-defective individuals who are fair-skinned, sun-sensitive and melanoma prone.

## 9. Oxidative Stress and Aging

**Figure 3:**
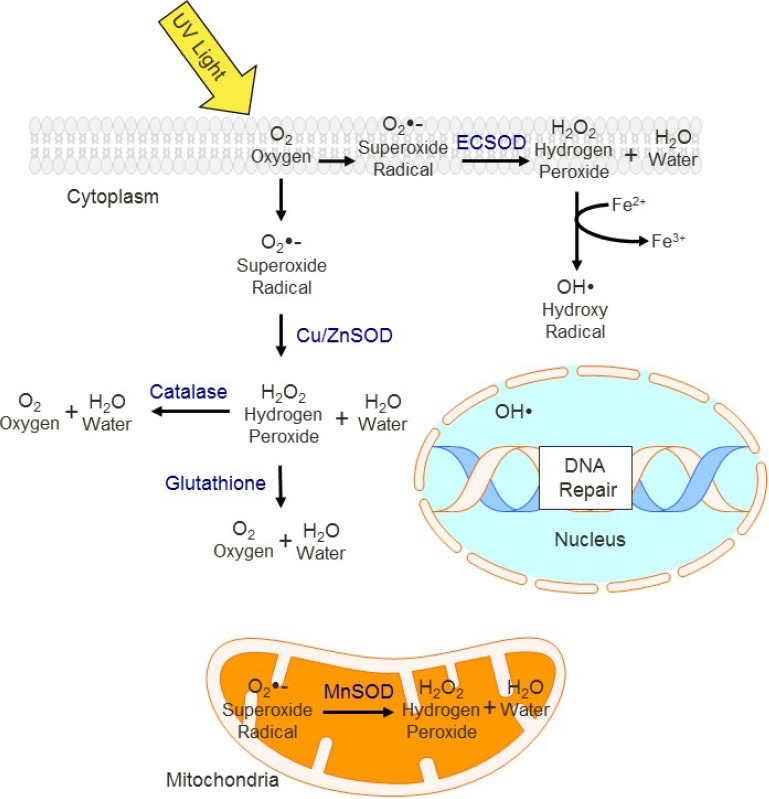
Cellular antioxidant defenses. UV induces a variety of free radical and oxidative molecules, which because of their chemical reactivity alter the molecular structure and damage lipids, proteins and nucleic acids [79]. Antioxidant enzymes mediate the removal of ROS, with different enzymes functioning in specific compartments (e.g., MnSOD localized to mitochondria). If not removed, ROS may react with DNA and other cell signal proteins, impairing their function. ECSOD, Extracellular Superoxide dismutase. Cu/Zn SOD, copper/zinc superoxide dismutase. MnSOD, Manganese Superoxide dismutase.

Reactive oxidative species (ROS) are produced by cells during normal metabolic activities such as mitochondrial oxidative phosphorylation, however levels of ROS vary with UV exposure and levels of antioxidant enzymes. Figure 3 shows a simplified scheme of the location of protective antioxidant enzymes in the cell (Figure 3).

Without inactivation, ROS damage macromolecules including lipid, proteins and DNA. UV, particularly longer-wavelength UVA, is a well-known inducer of ROS, and UV-induced oxidative stress may be an important contributive factor for melanoma [80,81,82]. ROS can inappropriately activate signaling pathways, interfere with genome maintenance and affect apoptosis. Numerous studies have tested the effects of solar radiation and oxidative stress on the skin [29,83,84,85], and oxidative stress has been linked to age-related loss of skin elasticity [86,87,88], defective cellular signaling [68] and photoaging [89,90]. Because it triggers cellular damage pathways, oxidative stress activates cellular senescence which is thought to directly lead to photoaging [91,92,93,94]. Cellular senescence is associated with a reduced capacity to divide and proliferate, sometimes in conjunction with shortening of telomeres [95,96,97,98]. Yokoo *et al.* found that exposing cells to a pro-oxidant agent (H_2_O_2_) impaired telomerase function which eventually resulted in telomere shortening, decreased proliferation and cellular enlargement [97]. Wrinkling of the skin is one of the most overt signs of photoaging, and UV exposure can induce wrinkling over time [99,100,101,102]. Though the molecular mechanism(s) of wrinkling are likely to be complex, UV exposure may reduce elastic properties of the skin to alter the three-dimensional structure of elastic fibers [103]. Using an animal model, Shin *et al.* noted an inverse correlation between wrinkling and important antioxidant enzymes that reduce cellular levels of ROS [104]. Thus UV-induced oxidative cutaneous damage may play a major role in photoaging.

Cells have a network of antioxidants and antioxidant enzymes that function to inactivate ROS and limit free radical injury. Because they house the enzymes that mediate the electron transport chain, mitochondria are the main intracellular source of endogenous levels of ROS. Manganese superoxide dismutase (MnSOD), a mitochondrial protein, is a major regulator of ROS in the mitochondria. Glutathione is the most widely expressed antioxidant in the cell, and its levels and oxidation state are regulated by feedback signaling dependent on total ROS level. Increases in ROS lead to use and depletion of glutathione and trigger recruitment of antioxidant enzymes such as catalase and superoxide dismutases (SODs).

Many synthetic and natural products have been reported to enhance levels of antioxidant enzymes, which make them therapeutic candidates to mitigate UV-mediated damage and to prevent the health consequences of UV exposure. Some products include alpha-tocopherol, selenium, phloretin, ferulic acid, flavangenol, lipoic acid, and uric acid [57,105,106,107,108,109,110,111] as well as a variety of flavonoids derived from plants including pomegranate and strawberry [112,113]. Lipid-soluble carotinoids such as lycopene and beta-carotene have been reported to scavenge superoxide radicals [114] and to promote vitamin A activity [115]. However, large doses of UV may inactivate carotenoids in the skin and promote degradation of dermal collagen and elastin [114,116]. Vitamin C is another anti-oxidant compound that has been studied as a UV photoprotective agent [108], particularly in combination with other compounds such as ferulic acid and phloretin [108].

There is emerging evidence implicating MC1R and cAMP signaling in regulating antioxidant proteins. Using keratinocytes transfected with MC1R, Henri *et al.* reported lower cellular levels of ROS after pharmacologic activation MC1R/cAMP pathway and higher levels of ROS when PKA was pharmacologically inhibited [117]. In other work using human melanocytes, Song and colleagues found that αMSH-induced MC1R signaling increased levels of catalase after UV exposure. Catalase is an antioxidant enzyme that converts excess of hydrogen peroxide molecules to water and molecular oxygen [118]. Finally, Kaderaro and coworkers reported that cAMP stimulation reduced levels of hydrogen peroxide, an important ROS, in human melanocytes after UV exposure [74]. Therefore, there is great interest in exploiting the MC1R UV-protective signaling pathway as a protective mechanism against UV-mediated oxidative injury.

## 10. Conclusions

UV exposure is one of the most important environmental health hazards, clearly causative for age-related skin changes such as wrinkling, pigmentary changes, thinning and carcinogenesis. Because of complex societal factors, UV exposure may actually be increasing through increased occupational and recreational activities including indoor tanning. As we learn more about innate signaling mechanisms that regulate natural antioxidant defense pathways in the skin such as the MC1R hormonal axis, new approaches are being designed to exploit these signaling pathways to delay or even prevent free-radical induced symptoms of aging. Use of natural extracts such as forskolin derived from the roots of the Plectranthus barbatus (Coleus forskolii) plant may enhance protection against UV-induce molecular damage to the skin. cAMP-induced melanin deposition and antioxidant induction may prove to be an important therapeutic opportunity to reduce UV-mediated pathologies.
